# Development and validation of the Comprehensive Cannabis Motives Questionnaire (CCMQ)

**DOI:** 10.1177/02698811251341371

**Published:** 2025-06-19

**Authors:** John Moffitt, Carl Roberts, Paul Christiansen

**Affiliations:** Department of Psychology, University of Liverpool, Liverpool, UK

**Keywords:** Cannabis motives, exploratory factor analysis, confirmatory factor analysis, measurement invariance, structural equation modelling

## Abstract

**Background:**

Existing scales that measure cannabis use motives have failed to incorporate the full range of motives that underpin cannabis consumption, especially with the increased use of medical cannabis. The current research aimed to develop a novel, psychometrically robust scale that comprehensively measures cannabis use motives. Here, we report the development and validation of the Comprehensive Cannabis Motives Questionnaire (CCMQ).

**Method:**

Cannabis users completed a 45-item questionnaire measuring a range of cannabis use motives. A UK English-speaking sample (*n* = 450) provided data for exploratory factor analysis. A second UK English-speaking sample (*n* = 200) was used for confirmatory factor analysis. Test–retest reliability was based on a third English-speaking sample (*n* = 45) who completed the revised, 41-item CCMQ twice across 2 weeks. A US-based sample (*N* = 216) was used to test measurement invariance of the scale across countries.

**Results:**

Exploratory and subsequent confirmatory factor analysis provided an eight-factor solution. The eight factors were food, medicinal, sleep, social, high, coping, conformity and creative. All the factors had good to excellent internal reliability with McDonald’s ω ranging between 0.85 and 0.97. Test–retest reliability was obtained for the revised 41-item questionnaire (Intraclass correlation’s 0.5+ for Total Cannabinoid Eating Experience Questionnaire and each subscale). The eight factors were correlated with Cannabis Use Disorder Identification Test – Revised to assess relationships with problematic use. Finally, strict measurement invariance was achieved in comparisons between males and females and a UK sample against a US sample.

**Conclusion:**

The CCMQ provided a valid, reliable assessment of the motivations that underlie cannabis use.

## Introduction

The United Nations Office on Drugs and Crime (UNODC) (2019) reports that 219 million people use cannabis globally. In England and Wales alone, it is estimated that two-and-a-half million people used cannabis within the last year, and cannabis was the most common substance that young people sought treatment for (Office for National Statistics, 2023a, 2023b). Various reasons for cannabis use have previously been identified; these include coping with negative moods (internal, negatively reinforcing), enhancing positive moods (internal, positively reinforcing), conforming to peer expectations (external, negatively reinforcing), facilitating social interactions (external, positively reinforcing) and expanding awareness (Simons et al., 1998). In November 2018, cannabis-based products were made legal for medicinal use (albeit for a limited number of conditions, such as Lennox-Gastaut syndrome, Dravet syndrome and chemotherapy-induced nausea; National Institute for Heath and Care Excellence, 2019). Despite this, NHS cannabis prescriptions remain scarce with only 12 prescriptions since 2018 (Schlag et al., 2020). Ramifications of a scarcity of NHS prescriptions mean that some patients have been able to access cannabis-based medicinal products through private prescriptions albeit at significant cost (Nutt et al., 2020) and for those unable to afford a private prescription Couch (2020) posited that there are over 1 million cannabis users self-medicating with street cannabis. The duality of cannabis’s propensity for abuse and therapeutic potential highlights the importance of understanding the underlying motives behind cannabis use.

Cannabis is made up of over 500 components (Pertwee and Cascio, 2014), with over 100 of them cannabinoids (Pagano et al., 2022). These cannabinoids interact with specific cannabinoid receptors, which are spread across the central and peripheral nervous system and are collectively known as the endocannabinoid system (ECS). The ECS has an essential role in several functions, including sleep, memory and reward signalling (Svíženská et al., 2008). As a result of this, cannabis consumption elicits a plethora of effects, each of which could be a potential motivating factor behind its ongoing use.

There have been previous attempts to measure the motives that underly cannabis use. Simons et al. (1998) utilised the motivational model of drug use, essentially adapting Cooper’s (1994) alcohol motives questionnaire. Cooper argued that drug use can be viewed using a motivational model of positive versus negative reinforcement, which can be further split into internal and external. Enhancement is an example of internal positive reinforcement (i.e. the feeling of being high) being the driving motivator behind the behavior. Coping is the internal negative reinforcement where drugs are used to cope with adverse effects (e.g. anxiety and depression). Social use is an example of external positive use (e.g. enjoying using drugs with friends), and conformity is an example of external negative use (e.g. using cannabis to fit in). Simons and colleagues also added a new additional factor called expansion. This included items such as ‘to know myself better’ and ‘to be more open to experiences’ which were a mix of adaptation of questions from work on cognitive motivation (Newcomb et al., 1988) and author-created questions. Using the 24-item Marijuana Motives Measure (MMM) Simons et al. (1998) found that cannabis use was best explained by a five-factor model corresponding to the previously discussed factors (Enhancement, Social, Conformity, Coping, and Expansion).

However, there are some limitations with the MMM and its development. Firstly, the nature of the sample (first-year American university students) limits the generalisability of the study (e.g. not including medical use – as older adults are more likely to use cannabis for medicinal purposes, Haug et al., 2017; Yang et al., 2021). Additionally, if the study were to follow current sample size recommendations 5–10 participants per item (Comrey and Lee, 2013), the study would be considered substantially underpowered for the type of analysis used. Despite subsequent confirmatory factor analysis indicating that the factor structure had a good fit, the fit statistics suggest an acceptable to poor fit (Zvolensky et al., 2007). Furthermore, in the study by Simons et al. (1998), there are substantial limitations in the assessment of predictive validity of the scale (e.g. square root transforming predictors, stepwise regression). Matali et al. (2018) translated the scale into Spanish and ran a test–retest analysis, in which the scale performed poorly (ICC’s; Coping = 0.40, Enhancement = 0.35, Expansion = 0.30, Social = 0.22, and Conformity = 0.001). Additionally, the timeline for the test–retest procedure was not specified, which limits our understanding of the stability and reliability of the measurements over time. The subscales of the MMM correlate with cannabis-related outcomes. For example, Benschop et al. (2015), found that the coping motives were associated with cannabis dependence. Chabrol et al. (2005) found that enhancement motives were a significant predictor of past 30-day cannabis use and that expansion motives were a predictor of dependent cannabis use assessed by DSM-IV criteria for cannabis dependence.

More recently, Lee et al. (2009) developed the Comprehensive Marijuana Motives Questionnaire (CMMQ). This scale is comprised of 12 factors, each consisting of 3 items. The factors are Enjoyment, Conformity, Coping, Experimentation, Boredom, Alcohol, Celebration, Altered Perceptions, Social Anxiety, Relative Low Risk, Sleep and Availability. Analysis of the CMMQ revealed positive correlations between all motives (except availability and alcohol) and cannabis use (assessed by ‘in the last 90 days, on how many days did you use any kind of marijuana or hashish?’). Additionally, correlations were found between all motives (except availability, boredom and enjoyment) and cannabis-related consequences (Rudgers Marijuana Problem Index; White et al., 2005)). Multiple regression found that low risk, enjoyment, sleep, boredom and altered perceptions were positively associated with cannabis use, whereas Experimentation and availability were negatively associated with cannabis use. Hierarchical regression was also performed and indicated that the factors sleep and coping were uniquely associated with more consequences, whereas enjoyment was associated with fewer problematic consequences. However, there are limitations with the analysis; one being that participant scores for the number of days cannabis was used in the previous 90, were capped at 45 to reduce the effect of outliers in the original analysis (Lee et al., 2009). Although the 12 factors are more expansive than those in the study by Simons et al. (1998), there are issues, including considerable overlap between factors, for example, Coping (e.g. ‘to forget about your problems’) and Social Anxiety (e.g. ‘o feel more confident’). Specific correlations between factors were not reported in the paper, however, they ranged between 0.13 and 0.73. Lee et al. (2009) also attempted to include items to address medicinal use; however, potentially due to a lack of diversity in the sample, the medicinal items were dropped following exploratory factor analysis (EFA). The exclusion of medicinal items may not reflect a flaw in the questionnaire itself but rather the limitations imposed by the homogeneity of the study sample. Given that first-year college students (mean age 18.1 ± 0.44) may have limited exposure to or experience with medicinal cannabis use, the sample was not ideally suited to comprehensively evaluate the inclusion of such items. This underscores the importance of testing psychometric tools in diverse populations to ensure broader applicability and relevance. CMMQ underwent further psychometric evaluation through confirmatory factor analysis and performed well. However, it is unclear how many participants were included in this analysis and whether this sample was separate from that of the EFA. Additionally, the CMMQ did not undergo test–retest reliability, so the consistency and stability of this measure over time are uncertain. Regarding how the factors have contributed to understanding cannabis-related behaviors, Bonn-Miller et al. (2014) found that, among medicinal cannabis users, all CMMQ motives (excluding relatively low risk, sleep and conformity) were associated with increased use. In contrast, Bohnert et al. (2018) found that sleep motives were associated with higher use, and coping was associated with lower mental health functioning. Blevins et al. (2016) found that conformity, coping and boredom were all correlated with negative consequences of cannabis use.

Although motivations for cannabis use have been previously studied (Lee et al., 2009; Simons et al., 1998), these questionnaires exhibit limitations in scope, psychometric rigour and applicability beyond their original context. Specifically, both instruments were developed using samples of first-year university students in the US, limiting their generalisability to broader populations, particularly those with diverse patterns of use or motivations, such as medicinal use. While the existing questionnaires performed adequately within their specific contexts, they do not fully capture the breadth of motivations for cannabis use in more diverse populations.

To address these gaps, we opted to develop a new questionnaire rather than directly adapting previous tools. This decision was driven by the need to incorporate novel constructs (e.g. motivations related to medicinal use) and to ensure rigorous psychometric validation across a broader and more representative sample. While informed by the strengths of prior work, our approach focuses on creating a tool designed to address the specific limitations of existing measures and to better reflect the diversity of cannabis use motivations in contemporary populations.

The current study aims to achieve this with the initial development and validation of ‘CCMQ’, which aims to assess a range of motives while being developed on a more representative cannabis-using population. This will be achieved by conducting parallel analysis, EFA, CFA and test–retest analysis. In addition to this, measurement invariance testing will be conducted to assess whether the questionnaire performs similarly in males and females, and the US versus UK sample. Finally, a structural equation modelling (SEM) will be employed to assess whether the factors identified are associated with problematic cannabis use. Given that the structure emerging from the factor analysis in the initial phase of the study was unknown, we refrained from hypothesising specific directional associations between the identified factors and Cannabis Use Disorder Identification Test – Revised (CUDIT-R); however, it was hypothesised that the factors would be associated with CUDIT-R scores.

## Methods

### Participants

In total, four samples – three from the UK and one from the US – were studied, and it was ensured that each analysis was supported by adequate sample sizes. For EFA, it is suggested that sample sizes have a minimum of 300 participants (Tabachnick et al., 2013). Additionally, Comrey and Lee (2013) suggest a ratio of 10 participants to 1 item in EFA as the minimum required. For confirmatory factor analysis, a multifaceted approach to sample size was used, as there is no single agreed-upon method. Firstly, a Monte Carlo power simulation was performed, this suggested a minimum sample size of 56 participants. In addition to this, a sample of 200 was targeted as this is suggested as a rule of thumb (Hoe, 2008; Singh et al., 2016) and is considered fair in a graded analysis by Comrey and Lee (2013). For factor analysis, the final samples were EFA (*N* = 450) and CFA (*n* = 200). A third sample (*n* = 45) was recruited for test–retest analysis (participants completing the scale identified by the EFA/CFA completing the questionnaire twice, 2 weeks apart). G*Power (Faul et al., 2007) was used to calculate the sample size needed for test–retest analysis (*n* = 45). The final sample consisted of participants from the US and was used for measurement invariance testing (*N* = 216). Demographic information can be seen in Table 1.

**Table 1.** Demographic information for all four samples.

Variable	Sample 1, *n* = 450 (%)	Sample 2, *n* = 200 (%)	Sample 3, *n* = 45 (%)	Sample 4, *n* = 216 (%)
Gender				
Male	201 (44.67%)	80 (40.00%)	25 (55.56%)	158 (73.15%)
Female	231 (51.33%)	111 (55.50%)	19 (42.22%)	48 (22.22%)
Other	15 (3.33%)	8 (4.00%)	1 (2.22%)	8 (3.70%)
PNTS	3 (0.67%)	1 (0.50%)	0 (0.00%)	2 (0.93%)
Age *M* (±SD)	24.46 (±9.04)	24.93 (±9.63)	23.62 (±6.60)	33.91 (±9.48)
Age range	18–66	18–68	19–40	18–70
Age of first use (±SD)	16.80 (±3.96)	16.67 (±4.06)	16.80 (±5.70)	21.30 (±6.08)
Medicinal Y/N	123/327	47/153	4/41	157/58
CUDIT-R mean score	10.81 (6.93)	9.85 (6.46)	9.67 (5.18)	N/A
CUDIT-R score				
12+	176 (39.11%)	70 (35.0%)	17 (37.78%)	
8+	275 (61.11%)	100 (54.5%)	24 (53.33%)	

Other/non-binary third gender. Age is recorded in years. One participant in sample 4 did not answer whether they were a medicinal user. No CUDIT-R data were collected for sample 4.

PNTS: prefer not to say.

Recruitment for all samples was conducted in the same manner, via online advertisements posted across social media platforms (e.g. Instagram, X and Facebook) and forum-based websites (e.g. Reddit and Bluelight.org). The study was hosted on the Qualtrics survey platform and advertised as an investigation into motivations for cannabis use. It was open only to adults (over 18 years of age) who had used cannabis at least once in the past 3 months. To determine whether participants used cannabis for medicinal purposes, they were asked, ‘Do you currently or have you ever used cannabis for medicinal purposes?’

The study was completed anonymously, and a bot check was implemented to prevent automated responses. Incentives varied by sample. Participants in Samples 1 and 2 were entered into a prize draw to win one of ten £10 Amazon vouchers. Participants in Sample 3 were offered a £20 Amazon voucher upon completion of a follow-up questionnaire. Sample 4 participants were offered the same incentive as those in Samples 1 and 2, but denominated in US dollars. Ethical approval was granted by the University of Liverpool Research Ethics Committee (reference number: 11679).

### Measures

#### Comprehensive Cannabis Motives Questionnaire

The CCMQ was developed to assess a wide range of motivations for cannabis use, encompassing eight subscales: Food, Medicinal, Sleep, Social Enhancement, Conformity, Coping, Aesthetic Enhancement and Cognitive Enhancement. These subscales were derived through a review of existing literature and consultations with cannabis users. Individuals who used cannabis were actively involved throughout the development process to ensure that the items were relevant, accurately worded and reflective of real-world experiences. All items were rated on a five-point Likert Scale (1 = strongly disagree to 5 = strongly agree).

Nineteen items were adapted from the Drinking Motives Questionnaire (Cooper, 1994), with five items each selected for the Social, Enhancement and Coping subscales, and four items for the Conformity subscale. One item originally designed to assess conformity (‘so that others won’t kid you about not drinking’) was excluded due to poor applicability in a UK sample and because the remaining four items sufficiently captured conformity-related motives.

Food-related motives were assessed using six items from the Hedonic Eating subscale of the Cannabinoid Eating Experience Questionnaire (Roberts et al., 2019). These six items were chosen based on the highest factor loadings reported by Roberts et al. (2019). The Appetitive subscale was excluded as it addressed more implicit processes that were not suitable for the self-report format of the CCMQ.

Sleep-related items were developed by adapting five of the seven components of the Pittsburgh Sleep Quality Index (PSQI; Buysse et al., 1989): subjective sleep quality, sleep latency, sleep duration, sleep disturbances and use of sleep medication. The components relating to daytime dysfunction and sleep efficiency were excluded due to difficulties in adapting them appropriately for cannabis use (e.g. ‘I use cannabis to make my sleep more efficient’) and the incompatibility of the PSQI’s Likert format with that of the CCMQ. Items were developed by rewording components into statements suitable for a standard Likert Scale (e.g. ‘I fall asleep quicker with cannabis’; ‘I have a less disturbed sleep with cannabis’). In total, five items assessed sleep-related motives.

There were no existing measures suitable for assessing medicinal cannabis use in a manner consistent with the CCMQ’s format. As such, nine items were created. Two focused on chronic pain (e.g. ‘I use cannabis for chronic pain [long term]’), and seven explored the use of cannabis in relation to prescribed medications (e.g. ‘I use cannabis to avoid some of the side effects of other medication[s]’).

Six further items were created to assess aesthetic and cognitive enhancement (e.g. ‘I use cannabis to improve my creativity’ and ‘I use cannabis to feel closer to nature’). All 45 items were randomised into a single presentation order, which was identical across participants to minimise order effects. Reverse scoring was not necessary, as items were intentionally worded in a consistent direction to reduce the risk of spurious factor emergence (Zhang et al., 2016).

#### Cannabis use disorder identification test – revised

The CUDIT-R (Adamson et al., 2010) is an eight-item screening tool designed to assess problematic cannabis use. Each item is rated on a five-point Likert Scale (0–4), yielding a total score ranging from 0 to 32. Scores between 8 and 11 indicate hazardous use, while scores of 12 or above suggest more problematic use and may indicate the presence of cannabis use disorder. The CUDIT-R was administered at the end of the survey to examine associations between cannabis use motivations and levels of problematic use.

#### Data reduction and statistical analyses

Only those participants who completed the full surveys were analysed. Data were analysed using RStudio (Version 4.2.0).

## EFA (sample 1)

Sampling adequacy of the CCMQ was assessed by the Kaiser–Meyer–Olkin (KMO) measure and Bartlett’s test of sphericity. KMO scores between 0.50 and 0.70 are considered acceptable, and values above 0.70 are considered good to excellent (Hutcheson and Sofroniou, 1999). Bartlett’s test of sphericity was performed to ensure that adequate correlations between items were used for EFA. To estimate the number of factors, a parallel analysis was performed (i.e. considered the best method for extracting factors from a dataset; Ledesma and Valero-Mora, 2007; Velicer et al., 2000). Following this, an EFA was conducted on the polychoric correlation matrix (due to the data being ordinal) to determine the underlying factor structure. An oblimin rotation was employed as the factors were expected to be correlated (Fabrigar et al., 1999). Items were removed from the scale if they had no factor loading above 0.5, or a loading of more than 0.5 on one item but also a loading of more than 0.32 on another factor (Costello and Osborne, 2005).

## Confirmatory factor analysis (sample 2)

The CFA was conducted in RStudio using the Lavaan package. The fit indices that were used for CFA included the standardised root mean residual (SRMR), with values under 0.08 being indicative of good fit, the Tucker–Lewis index (TLI) and Comparative Fit Index (CFI) with acceptable fit judged at >0.90 and good at >0.95 (Hu and Bentler, 1999). Finally, the RMSEA parsimony adjusted measure is reported with values <0.06 being a good fit and values >0.06 but <0.08 being acceptable (Hu and Bentler, 1999). The Diagonally Weighted Least Squares (DWLS) estimator was used due to the dataset being ordinal (Mindrila, 2010) and the Likert Scale data being heavily skewed (Ghosh et al., 2018).

## Test retest reliability (sample 3)

Interclass correlation analysis was used to assess test–retest reliability. Values > 0.6 indicate good test–retest reliability (Cicchetti, 1994).

## SEM (samples 1 and 2)

SEM was performed to assess relationships between the identified factors and the CUDIT-R. Factors were regressed onto the total CUDIT-R (Adamson et al., 2010) score, as this allowed the assessment of the factors’ impact on the CUDIT-R score without any error variance. The method of estimation was DWLS, and model fit indices were the same as previously discussed.

## Bivariate correlations (samples 1 and 2)

In addition to conducting SEM, bivariate correlations were calculated between subscales and the CUDIT-R. These analyses aimed to examine the direct relationships between individual subscales and cannabis use severity as measured by the CUDIT-R.

## Measurement invariance (samples 1, 2, and 4)

To ensure the scales performed consistently between males and females, and between US and UK samples, measurement invariance testing was conducted. The first step in this process was to assess configural invariance (whether the factor structure holds across two samples) by fitting the factor structure identified with a grouping variable (sex or country), which was evaluated using the same fit indices previously discussed for CFA and SEM (CFI, RMSEA and SRMR). Following this, we compared the configural model to a metric model (fixing factor loadings across groups while allowing intercepts to vary). This comparison determined whether each item contributed to the factor similarly across the groups and was assessed by comparing the fit indices between the two models with differences of ∆CFI < 0.01, ∆RMSEA < 0.015 and ∆SRMR < 0.03 as the cut-offs (Chen, 2007) for showing metric invariance. Next, the metric invariance model was compared to the scalar invariance model, which assumes equal factor loadings and intercepts across groups, allowing for the comparison of factor means across groups. The assessment for scalar invariance was similar to that for metric invariance, except the SRMR cut-off was stricter at <0.015. Finally, we also examined strict invariance, in which residuals, slopes and intercepts were assumed to be constant, to determine if the items’ unique variances were consistent across groups. This model was compared to the scalar invariance model using the same cut-off values as in the previous comparison.

## Internal reliability and descriptives (samples 1, 2 and 4)

The internal consistency of each factor was calculated by McDonald’s omega (ω) total (see Revelle and Zinbarg, 2009); it does not assume tau equivalence and is not a lower bound estimate. Internal consistency of the full scales was assessed by ω hierarchical (i.e. the reliability of an overarching factor ‘g’). Values greater than 0.7 are deemed acceptable (McNeish, 2018).

## Results

Information on the type of cannabis use for all samples can be seen in Table 2.

**Table 2.** Cannabis use information for all four samples.

Variable	Sample 1	Sample 2	Sample 3	Sample 4
Form used				
Flower	407 (90.44%)	185 (92.5%)	37 (77.8%)	194 (89.8%)
Edibles	27 (6%)	10 (5%)	2 (4.4%)	11 (5.1%)
Oil	9 (2%)	2 (1%)	1 (2.2%)	10 (4.6%)
Hash/Resin	7 (1.56%)	3 (1.5%)	5 (4.4%)	1 (0.5%)
Last use				
Last 24 h	214 (47.56%)	96 (48%)	24 (53.3%)	88 (40.7%)
Last week	96 (21.33%)	40 (20%)	9 (20%)	103 (47.7%)
Last month	78 (17.33%)	34 (17%)	7 (15.6%)	16 (7.4%)
Last 3 months	62 (13.78%)	30 (15%)	5 (11.1%)	9 (4.2%)

Flower is a combination of Indica, Sativa, hybrid and skunk. This was done because, while cannabis is illegal in the UK, participants cannot be sure what strain they are consuming.

### Parallel analysis (sample 1)

A parallel analysis initially identified that there were potentially 10 factors. After establishing an upper limit of 10 factors, subsequent EFAs with oblimin rotations were run, working back from 10 to assess which factor solution best fit the data.

### EFA (sample 1)

The sampling adequacy was determined to be excellent (KMO = 0.91), and Bartlett’s test of sphericity indicated that correlations between items were sufficient for EFA (χ^2^ (990) = 15542.95, *p* < 0.001). Following the removal of items (as described in the section ‘Methods’), an eight-factor solution with 41 items was finalised, explaining 65% of the total variance (see Table 3 for factors, their Eigenvalues and variance explained).

**Table 3.** Eigenvalues for each factor identified through EFA.

Factor	Eigenvalue	% of variance	Cumulative variance (%)
1. Food	5.35	12%	12
2. Medicinal	5.18	12%	24
3. Sleep	4.25	9%	33
4. Social	3.12	7%	40
5. High	3.07	7%	47
6. Conformity	2.76	6%	53
7. Coping	2.67	6%	59
8. A&C Enh	2.63	6%	65

Eigenvalues for each factor alongside the factor name. A&C Enh is a shortened form of aesthetic and cognitive enhancement.

Factor one consisted of six items and was called food. Factor two was comprised of nine items and was called medicinal. Factor three comprised of five items and was called sleep. Factor four had four items and was called social. Factor five consisted of four items and was called high. Factor six comprised of five items and was called conformity. Factor seven comprised of four items and was called coping. Finally, factor eight consisted of five items and was called aesthetic and cognitive enhancement. Four items were dropped for not having a loading >0.5 on any of the factors. Items within all factors are summarised in Table 4 with factor correlations shown in Table 5. Factors were relatively independent with factor correlations ranging between −0.20 and 0.46.

**Table 4.** Factor loadings and communalities for oblimin-rotated eight-factor solution for 45 CCMQ items (*N* = 450).

I use cannabis. . .	Factors								
	1	2	3	4	5	6	7	8	Comm
To make food taste better	**0.94**	0.02	0.02	0.01	−0.01	−0.01	0.00	−0.02	0.90
To make food more delicious	**0.89**	−0.02	0.02	−0.01	0.06	0.01	0.00	0.04	0.87
To make tastes and flavours more intense	**0.88**	−0.02	−0.02	0.02	−0.03	−0.02	0.04	0.01	0.78
To like food more	**0.86**	0.06	−0.01	0.01	−0.05	−0.02	0.05	0.02	0.75
To make the experience of eating food better	**0.85**	0.07	0.04	−0.06	0.07	0.02	−0.01	0.02	0.80
To make flavours more complex	**0.79**	−0.01	0.05	0.05	−0.01	−0.01	0.01	0.03	0.69
Because it is more effective than other medications	−0.03	**0.82**	0.05	0.01	0.00	−0.04	−0.02	0.08	0.76
To avoid some of the side effects of other medications	0.01	**0.79**	−0.01	0.00	−0.03	−0.07	−0.04	−0.06	0.63
Because the side effects are preferable to other medications	−0.08	**0.74**	0.04	−0.04	−0.04	0.03	0.07	0.11	0.67
When I’m unwell, because I prefer the method of intake (smoke/vape/edible) rather than pills	0.09	**0.74**	0.03	−0.07	0.08	−0.10	−0.02	−0.05	0.57
To help with chronic pain (long term)	−0.02	**0.70**	0.02	−0.01	−0.16	−0.08	0.10	−0.02	0.66
Because it is easier to gauge the correct dose compared to conventional medication when ill	0.12	**0.70**	0.00	0.04	0.03	0.12	−0.13	0.08	0.50
To help with acute pain (short term)	0.07	**0.69**	0.02	−0.02	−0.06	−0.09	0.09	−0.02	0.60
Because it makes prescribed/legal medication(s) more effective	0.02	**0.58**	0.01	0.03	−0.04	0.09	0.08	0.08	0.41
Because it is more convenient than legal medication	0.11	**0.58**	−0.01	0.04	0.09	0.09	0.08	−0.02	0.36
To have a better night’s sleep	0.02	−0.03	**0.97**	−0.03	0.01	0.01	−0.04	0.00	0.89
To help you sleep	−0.04	−0.02	**0.97**	−0.01	−0.01	0.00	−0.01	0.01	0.91
To have a less disturbed sleep	0.00	0.03	**0.91**	0.02	0.02	−0.06	−0.01	−0.05	0.83
To fall asleep quicker	0.02	0.01	**0.82**	−0.03	−0.03	−0.02	0.08	0.02	0.77
To sleep longer	0.09	0.03	**0.72**	0.06	−0.03	0.07	0.06	0.02	0.63
To improve parties and celebrations	0.00	−0.03	−0.06	**0.89**	−0.03	−0.07	0.05	0.02	0.78
To help you enjoy a party	0.00	−0.01	0.03	**0.84**	0.01	0.02	−0.01	0.00	0.72
To make social gatherings more fun	0.05	−0.03	0.01	**0.71**	0.15	0.07	−0.01	0.02	0.70
To be more sociable	−0.05	0.21	0.06	**0.51**	0.02	0.19	−0.03	0.03	0.38
Because it is fun	0.08	−0.04	−0.03	0.10	**0.81**	0.07	−0.09	0.00	0.80
Because I like the feeling of being high	−0.05	−0.03	0.01	0.02	**0.80**	−0.10	0.11	0.06	0.72
Because it gives a pleasant feeling	−0.04	0.12	0.01	0.04	**0.66**	−0.08	0.19	0.06	0.57
To get high	0.08	−0.22	−0.03	0.01	**0.52**	0.00	0.05	0.08	0.46
To not feel left out	−0.02	−0.03	−0.02	−0.06	0.04	**0.85**	−0.03	−0.02	0.72
To fit in with a group you like	−0.04	−0.04	0.01	0.04	0.02	**0.81**	0.02	0.02	0.69
To be liked	−0.06	0.04	0.02	0.14	−0.07	**0.73**	0.08	0.02	0.61
Because your friends pressure you to smoke	0.11	−0.05	−0.07	−0.08	−0.09	**0.70**	−0.01	−0.03	0.50
To help you forget your worries	0.05	−0.04	−0.01	0.02	0.03	−0.01	**0.81**	0.03	0.70
To forget about your problems	0.08	−0.07	0.02	0.07	0.06	0.08	**0.77**	−0.05	0.69
To help you when you feel depressed or nervous	−0.05	0.14	0.08	−0.03	−0.05	−0.04	**0.76**	0.04	0.70
To cheer you up when you are in a bad mood	0.12	0.09	0.11	−0.01	0.21	0.03	**0.52**	0.06	0.57
To feel closer to nature	0.02	0.09	0.01	−0.04	0.06	0.07	0.08	**0.66**	0.56
To improve my creativity	−0.11	0.17	0.03	0.05	0.04	−0.12	−0.04	**0.63**	0.48
To make music sound better	0.24	−0.16	−0.03	0.09	−0.07	−0.10	0.10	**0.61**	0.57
To engage in deeper thoughts	−0.01	0.03	0.03	0.00	0.19	0.13	0.07	**0.59**	0.52
To make art look better	0.25	0.05	0.08	0.11	−0.05	0.04	−0.10	**0.58**	0.60
Because it is exciting	0.20	−0.13	−0.05	0.11	0.46	0.21	0.07	−0.05	0.53
To make films more enjoyable	0.29	−0.11	0.07	0.03	0.19	−0.05	−0.06	0.37	0.46
To celebrate a special occasion with friends	0.17	−0.11	−0.01	0.45	0.31	−0.01	−0.03	−0.01	0.54
To feel more self-confident and surer of myself	−0.17	0.25	0.15	0.37	0.00	0.16	0.20	0.13	0.46

Table includes all questions including the items dropped. Bold indicates a significant loading.

Comm: communality.

**Table 5.** Pearson’s correlations among extracted factors after oblimin rotation.

Factor	1	2	3	4	5	6	7	8
1. Food	—							
2. Medicinal	0.04	—						
3. Sleep	0.29	0.44	—					
4. Social	0.24	−0.06	0.02	—				
5. High	0.35	−0.26	−0.02	0.46	—			
6. Conformity	0.01	−0.21	−0.12	0.29	0.08	—		
7. Coping	0.22	0.24	0.40	0.27	0.27	0.04	—	
8. A&C Enh	0.46	0.29	0.29	0.38	0.30	0.02	0.28	—

A&C Enh: aesthetic and cognitive enhancement.

Eigenvalues can be seen in Table 3.

### Internal reliability and descriptive statistics – samples 1, 2 and 4

Table 6 shows that each of the eight factors had good to excellent internal reliability. However, the overall reliability of the full scale for sample one was below 0.7 (Hierarchical ω = 0.64) but, it is not envisaged that the full-scale score would be used. However, for sample two, the Hierarchical ω = 0.76, and sample four had a Hierarchical ω = 0.72.

**Table 6.** McDonalds ω for samples 1, 2 and 3

Factor	Sample 1 (EFA)	Sample 2 (CFA)	Sample 4 (US)
Food	0.97	0.97	0.88
Medicinal	0.93	0.93	0.84
Sleep	0.96	0.97	0.83
Social	0.87	0.9	0.74
High	0.88	0.85	0.77
Conformity	0.89	0.91	0.83
Coping	0.89	0.91	0.75
A&C Enh	0.85	0.85	0.77

A&C Enh: aesthetic and cognitive enhancement.

### CFA – sample 2

In total, 41 items were free to load on one of eight factors. The eight-factor model provided a good fit to the data on all fit indicators (χ^2^(751) = 757.02, TLI = 0.999, CFI = 1.000, SRMR = 0.070, RMSEA = 0.006).

Mean CCMQ total, subscale scores and *T*-tests for comparisons between samples 1 and 2 are summarised in Table 7.

**Table 7.** Total scale and subscale scores, *t*-tests (Welch) and effect size between samples 1 and 2.

Factor	S1 (EFA)	S2 (CFA)	t (df)	Cohen’s d
1. Food	19.31 (7.51)	18.40 (7.30)	1.46 (392.15)	0.12
2. Medicinal	22.5 (9.54)	21.30 (9.32)	1.51 (389.74)	0.13
3. Sleep	19.31 (5.60)	18.35 (6.15)	1.90 (351.83)	0.17
4. Social	12.38 (4.42)	11.84 (4.39)	1.48 (384.64)	0.13
5. High	16.86 (3.26)	16.90 (3.01)	−0.12 (411.69)	0.01
6. Conformity	7.18 (3.58)	6.83 (3.61)	1.15 (378.68)	0.09
7. Coping	14.59 (4.32)	13.76 (4.55)	2.12 (364)***	0.18
8. A&C Enh	14.99 (5.03)	15.33 (4.99)	−0.79 (386.66)	0.06
Total CCMQ	127.11 (24.71)	122.68 (26.03)	−2.04 (364.47)***	0.18

A&C Enh: aesthetic and cognitive enhancement; S1 and S2: sample 1 and sample 2.

*** p < 0.05.

#### Test re-test – sample 3

Six of the eight subscales showed good intraclass correlations (see Table 8). Two factors were borderline passes: High (0.53) and Conformity (0.56).

**Table 8.** Intraclass correlations (ICC) between times for sample 3.

Factor	ICC	p
1. Food	0.87	0.001
2. Medicinal	0.87	0.001
3. Sleep	0.86	0.001
4. Social	0.82	0.001
5. High	0.53	0.001
6. Conformity	0.56	0.001
7. Coping	0.76	0.001
8. A&C Enh	0.88	0.001
CCMQ total	0.86	0.001

A&C Enh: aesthetic and cognitive enhancement.

### CCMQ scores and CUDIT-R: Predicting cannabis use using subscales

The final model was a good fit for the data (χ2(1091) = 3466.91, TLI = 0.951, CFI = 0.948, SRMR = 0.068, RMSEA = 0.058).

Direct associations between the factors and mean CUDIT-R scores are shown in Table 9. Aesthetic and Cognitive Enhancement is the only factor to have no correlation. Food, Medicinal, High, Conformity and Coping all positively correlate, and Sleep and Social both negatively correlate with CUDIT-R.

**Table 9.** Regression coefficients for SEM model.

Regression	B (SE)	p	95% CI
Food → CUDIT-R	0.16 (0.03)	<0.001	0.11 to 0.21
Medicinal → CUDIT-R	0.20 (0.03)	<0.001	0.14 to 0.27
Sleep → CUDIT-R	−0.07 (0.03)	0.024	−0.13 to −0.01
Social → CUDIT-R	−0.15 (0.06)	0.004	−0.26 to −0.05
High → CUDIT-R	0.24 (0.07)	0.002	0.9 to 0.39
Conformity → CUDIT-R	0.14 (0.04)	0.001	−0.19 to −0.09
Coping → CUDIT-R	0.38 (0.03)	<0.001	0.30 to 0.46
A&C Enh → CUDIT-R	0.01 (0.06)	0.818	−0.14 to 0.11

A&C Enh: aesthetic and cognitive enhancement.

Results for the correlations between subscales and CUDIT-R can be seen in Table 10.

**Table 10.** Correlation coefficients for bivariate correlations.

Correlation	*r_s_*	p
Food → CUDIT-R	0.34	<0.001
Medicinal → CUDIT-R	0.29	<0.001
Sleep → CUDIT-R	0.27	<0.001
Social → CUDIT-R	0.06	0.141
High → CUDIT-R	0.22	<0.001
Conformity → CUDIT-R	−0.08	0.04
Coping → CUDIT-R	0.41	<0.001
A&C Enh → CUDIT-R	0.28	<0.001

A&C Enh: aesthetic and cognitive enhancement.

### Measurement invariance

Differences in fit indices were minimal and did not exceed the previously stated cut-off, demonstrating measurement invariance for sex and country (US vs UK sample). Fit indices can be seen in Table 11, and model change can be seen in Table 12.

**Table 11.** Model fit measures for structural invariance.

	RMSEA	CFI	TLI	SRMR
Sex	0.047	0.970	0.967	0.064
Country	0.049	0.967	0.971	0.065

For the sex invariance test, samples 1, 2 and 4 were combined; for the country, samples 1 and 2 were combined and compared to sample 4.

**Table 12.** Metric, scalar and strict invariance by groupings.

	Sex	Country
Metric		
∆RMSEA	0.001	0.001
∆CFI	−0.002	−0.003
∆TLI	−0.001	−0.002
∆SRMR	0.001	0.001
Scalar		
∆RMSEA	0	0
∆CFI	0	−0.001
∆TLI	0.001	0
∆SRMR	0	0.001
Strict		
∆RMSEA	0	0.003
∆CFI	−0.001	−0.006
∆TLI	−0.001	−0.005
∆SRMR	0.002	0.004

Table shows the change in model fit indices.

## Discussion

Following EFA, CFA and reliability analysis, an 8-factor, 41-item CCMQ was finalised. The subscales were food, medicinal, sleep, social, high, conformity, coping and aesthetic and cognitive enhancement. The CCMQ was shown to have a robust factor structure, and all subscales had excellent internal reliability. Two of the subscales (conformity and high) had ICCs just above 0.5, although all the other subscales showed excellent test–retest reliability. Seven of the subscales were associated either positively (food, medicinal, high, conformity and coping) or negatively (sleep and social) with problematic cannabis use, whereas aesthetic and cognitive enhancement was not.

The eight-factor solution was identified through EFA and supported by CFA. Food was comprised of six items. Medicinal consisted of nine items. Sleep consisted of five items. Social, high, coping and conformity each had four items. Finally, aesthetic and cognitive enhancement had five items. Six subscales had good to excellent internal consistency and test–retest reliability, although two (conformity and high) had test–retest ICCs just over 0.5. A potential explanation for this is that the two factors each had four items, making them more sensitive to changes than a factor with more items. The measurement invariance testing demonstrated that the CCMQ achieved metric, scalar and strict invariance across both sex differences and differences between the UK and the US. This indicates that the factor structure is robust across these groups, supporting the validity of subscale comparisons. It is worth noting, as highlighted in the results, that the use of subscales is recommended over the total score. While score ranges for each subscale are not yet established, future work will aim to define these ranges to further enhance the interpretability and practical utility of the measure. Taken together, these findings suggest that the CCMQ is a reliable and robust tool for assessing cannabis use motives.

Medicinal is one of the two motives not reflected in either of the previous scales (Lee et al., 2009; Simons et al., 1998). A possible explanation for this is the samples used. Lee et al. (2009) did include items related to medicinal use; however, potentially due to a young sample, these items did not survive EFA. Older people who use cannabis are more likely to use cannabis for medicine (Haug et al., 2017; Yang et al., 2021) meaning it was always unlikely that Lee’s sample would support these items. There is some overlap in using cannabis for sleep and medicine, however, the psychometric validation of the CCMQ supports the idea that both sleep and medication are independent motives. Sleep-motivated cannabis use was also reflected in the CMMQ (Lee et al., 2009) but not the MMM (Simons et al., 1998). Food is the other motive not found in the previous two scales. This is possibly because of the greater understanding of the effect of cannabis on appetitive drive and hedonic eating since these papers were published (Davies-Owen et al., 2025; Roberts et al., 2019).

Social is one of the four factors adapted from Cooper’s (1994) four-factor model supported in the CCMQ and also the MMM. In the CMMQ, ‘celebration’ is not too dissimilar to the CCMQ ‘Social’ factor, in that it includes items such as ‘because it was a special occasion’; however, no other items address social use in the ‘celebration’ subscale of the CMMQ. The CMMQ does include a factor named ‘social anxiety’; however, this is subsumed by ‘Coping’ in the current factor structure. The final motive identified in the CCMQ was aesthetic and cognitive enhancement. This contains items similar to the final motive (expansion) for the MMM (i.e. because it helps me be more creative and original). Additionally, this is also like the altered perception factor from the CMMQ. The novel factor of ‘high’ in the current work is not dissimilar to Cooper’s original ‘Enhancement’ factor from her four-factor model. Another factor derived from Cooper’s scale work is Coping, and this factor is also consistent with both the MMM and the CMMQ.

SEM and bivariate correlations were conducted to further explore the relationships between motives for cannabis use and problematic use as measured by the CUDIT-R. While SEM identified significant associations for all motives except aesthetic and cognitive enhancement, bivariate correlations provided additional insights into the nuances of these relationships. These complementary approaches highlight the value of using both multivariate and bivariate analyses to capture the complexity of these relationships and underscore the importance of considering potential confounding variables in SEM.

The current study found a positive relationship between food-motivated cannabis use and problematic cannabis use in both SEM and bivariate correlations. A potential explanation for this positive relationship is that if users were using cannabis around mealtimes, then they incidentally score higher on CUDIT-R due to increased time spent high, regular use (each mealtime) and time devoted to obtaining cannabis. This may be due to flavours being more intense or delicious and not wanting to revert to a less rewarding experience.

Medicinal was also positively correlated with problematic cannabis use. It is however notable that the CUDIT-R may not be suitable to assess problematic cannabis use in a medicinal using population. This is primarily due to the frequency with which cannabis-based medicines must be taken. Indeed, Loflin et al. (2018) argued that the CUDIT-R was not suitable in screening for cannabis use disorder in military veterans for this reason. Additionally, Sagar et al. (2021) found that removing items pertaining to frequency of use and ‘thoughts about cutting down’ increased the overall alpha of CUDIT-R in medicinal users. Both studies took place in the US, which has more progressive cannabis policies than the UK. Despite a scarcity of NHS prescriptions and expensive private prescriptions, the concept of cannabis as a medicine has gained traction in the UK. Moreover, people may be self-medicating with street cannabis and not dosing correctly and not following routes of administration that are optimal for specific medical conditions.

Sleep-motivated use was one of the two motives negatively associated with problematic cannabis use according to SEM. This finding could be partially attributed to the scoring of the CUDIT-R. For example, items assessing time spent ‘stoned’ might under-represent problematic patterns for sleep-motivated users if cannabis is consumed shortly before going to sleep, as time asleep could be conflated with time spent high. Similarly, the item asking how often users are unable to stop smoking once started may not align with the typical patterns of sleep-motivated use. Despite these nuances, SEM suggested lower CUDIT-R scores for individuals using cannabis for sleep. In contrast, bivariate correlations revealed a positive relationship between sleep-motivated use and problematic cannabis use. This discrepancy may reflect differences between SEM, which accounts for confounding variables, and bivariate correlations, which do not. Alternatively, it may highlight a subset of sleep-motivated users whose frequent use could be classified as problematic.

Short-term cannabis use can reduce sleep latency (Gorelick et al., 2013); however, long-term use may decrease total sleep duration due to tolerance effects. Previous studies also show mixed results: Bonn-Miller et al. (2014) reported no association between sleep motives and increased use, while Bohnert et al. (2018) found a positive association, potentially reflecting tolerance-driven escalation. While the current study does not fully reconcile these findings, it underscores the complexity of sleep-motivated cannabis use and its potential risks. The distinction between increased use and problematic use is crucial, as even non-problematic patterns according to the CUDIT-R can pose long-term health consequences. Social use was also negatively associated with problematic use. Social users are likely to be motivated to use cannabis on special occasions or in the presence of specific people, which reduces the frequency of their consumption and could explain the negative association. However, the bivariate correlation for social use was not significant, suggesting that this relationship may be less robust when examined without accounting for confounding variables. Social use was related to 30-day cannabis use through hierarchical multiple regression in the study by Bonn-Miller et al. (2014). These findings suggest that social contexts have a strong influence on excessive use.

Using cannabis to get high had the second strongest association with CUDIT-R scores, possibly reflecting a pattern of more frequent or intensive use. The bivariate correlation also showed a positive and significant relationship, aligning with the SEM results. In the CMMQ (Lee et al., 2009), the factor most similar to high was enjoyment, this factor was associated with increased use in the study by Bonn-Miller et al. (2014). In Simons et al. (1998), MMQ high was called enhancement, and this factor is a significant predictor of past 30-day cannabis use (Benschop et al., 2015).

Conformity was found to be positively correlated with problematic cannabis use according to SEM. However, the bivariate correlation revealed a small but significant negative relationship, suggesting that the association may not be as straightforward, when confounding variables are considered. This contrast highlights the complexities of examining conformity as a motive for cannabis use. Conformity was not found to be associated with increased use in the study by Bonn-Miller et al. (2014), but it was linked to negative consequences of cannabis use in the study by Blevins et al. (2016). Using cannabis to cope had the strongest correlation with problematic use in both SEM and bivariate correlations, this is something that would be expected as coping motives are robust predictors of substance-related problems (Cooper, 1994). Coping-motivated cannabis use has been shown to be associated with increased cannabis use (Bonn-Miller et al., 2014) and with negative consequences of cannabis use (Blevins et al., 2016). However, in the study by Benschop et al. (2015), it was found that coping was not a predictor of cannabis use in the last 30 days, possibly highlighting the issues with self-reported cannabis use or reflecting a period without any life stressors. Although SEM did not find aesthetic and cognitive enhancement to be significantly correlated with problematic cannabis use, the bivariate correlation revealed a moderate positive relationship. This discrepancy suggests that while SEM did not identify a significant association, the bivariate analysis highlights a potential connection between these motives and problematic use, warranting further exploration. Additionally, the findings from this study and previous studies allow a greater understanding of the behaviors of people who use cannabis to cope.

### Limitations

There are several limitations to the current study. The legal status of cannabis in the UK created a reliance on online recruitment for the sample and ruled out cannabis users without smartphone/internet access. Implications of the reliance on online recruitment may include that participants are particularly motivated to respond to cannabis research due to a general positivity toward cannabis. The current paper did ask about the preferred method of consumption. However, all analysis was based on generic cannabis use (CUDIT scores). Method of intake (inhalation and ingestion) and different preparations (cannabis–tobacco mixture) were not controlled for in this paper’s analysis. Focusing on a sample that only uses cannabis and a sample that uses a 50/50 mixture with tobacco may elicit different motivations. Furthermore, this study did not collect BMI data, primarily due to participant dropout when presented with this question. The lack of this data does not allow any analysis regarding BMI, food intake (as per Roberts et al., 2019) and different motives. Finally, Bossong et al. (2014) highlight the association between cannabis use and memory function; as the data in this paper is based on retrospective accounts of cannabis use, the reliability of these accounts is a known limitation in research of this nature. This issue may be particularly pronounced in the present study, given that approximately half of the sample reported using cannabis in the 24 h prior to assessment. This recent use could influence both memory function and the accuracy of retrospective reports. However, this reliance on retrospective data also offers the benefit of capturing real-world patterns of cannabis use, which can provide valuable insights into how individuals recall and report their substance use in naturalistic settings. We conducted exploratory analyses comparing subscale scores across four cannabis use recency groups (24 h, last week, last month, last 3 months; see Supplemental File 1). Individuals who had used cannabis more recently reported significantly higher endorsement of motives related to Sleep, Medicinal, Coping, Conformity, Aesthetic and Cognitive Enhancement, and Food, with no significant differences observed for Social or High motives. While these findings support the construct validity of the CCMQ, we acknowledge the risk of obfuscation posed by the possible cognitive effects of acute cannabis use, which may subtly influence how respondents engage with items. Future research could explore the effects of cannabis use recency and responses to items and attempt to disentangle acute from chronic effects further.

A limitation of this study is that the sample was drawn from the general population, and as such, the new instrument requires further validation in clinical mental health and addictive populations. However, the results of the CUDIT-R suggest that the samples in this analysis included a diverse range of substance use patterns, with samples 1, 2 and 3 each having at least 35% of participants scoring 12 or above on the CUDIT-R, indicative of a substance use disorder. Additionally, all samples had over 53% of participants scoring 8 or above, indicating hazardous use. Although the fourth sample did not include CUDIT-R data, the findings from the other samples suggest a substantial proportion of individuals with problematic substance use, partially addressing the concern about the lack of clinical or addictive populations.

### Recommendations

Regarding future research, further development of the CCMQ is critical to ensure its validity and reliability. This may include adapting the questionnaire in different languages and conducting more measurement invariance testing. Future research will focus on developing a short-form version of the CCMQ to improve usability, given its current length of 41 items across 8 factors. In addition, further research into the factors identified as risk factors for problematic use is encouraged. Research into the various motivations for using cannabis may provide insights for future development of effective interventions for individuals with cannabis use disorders. In addition, the findings of this study may help to inform public health messaging around cannabis use and harm reduction.

## Summary and conclusion

The CCMQ has provided a reliable set of motivations for cannabis use. Furthermore, the CCMQ is the most extensive and detailed attempt to detail the multiple different motivations for cannabis to date. The data gathered in this study identify eight motivational factors that contribute to cannabis use. Novel motivations for using cannabis identified in the current analysis include food, medicinal and sleep purposes, along with confirming existing motivations identified in previous work. These findings should provide solid foundations for future scale work assessing motivations for cannabis use.

## Supplemental Material

sj-docx-1-jop-10.1177_02698811251341371 – Supplemental material for Development and validation of the Comprehensive Cannabis Motives Questionnaire (CCMQ) Supplemental material, sj-docx-1-jop-10.1177_02698811251341371 for Development and validation of the Comprehensive Cannabis Motives Questionnaire (CCMQ) by John Moffitt, Carl Roberts and Paul Christiansen in Journal of Psychopharmacology

## References

[bibr1-02698811251341371] Adamson SJ Kay-Lambkin FJ Baker AL , et al. (2010) An improved brief measure of cannabis misuse: the Cannabis Use Disorders Identification Test-Revised (CUDIT-R). Drug Alcohol Depend 110: 137–143. 20347232 10.1016/j.drugalcdep.2010.02.017

[bibr2-02698811251341371] Benschop A Liebregts N van der Pol P , et al. (2015) Reliability and validity of the marijuana motives measure among young adult frequent cannabis users and associations with cannabis dependence. Addict Behav 40: 91–95. 25240105 10.1016/j.addbeh.2014.09.003

[bibr3-02698811251341371] Blevins CE Banes KE Stephens RS , et al. (2016) Change in motives among frequent cannabis-using adolescents: Predicting treatment outcomes. Drug Alcohol Depend 167: 175–181. 27577862 10.1016/j.drugalcdep.2016.08.018 PMC5037028

[bibr4-02698811251341371] Bohnert KM Bonar EE Arnedt JT , et al. (2018) Utility of the comprehensive marijuana motives questionnaire among medical cannabis patients. Addict Behav 76: 139–144. 28820970 10.1016/j.addbeh.2017.08.001 PMC5772711

[bibr5-02698811251341371] Bonn-Miller MO Boden MT Bucossi MM , et al. (2014) Self-reported cannabis use characteristics, patterns and helpfulness among medical cannabis users. Am J Drug Alcohol Abuse 40: 23–30. 24205805 10.3109/00952990.2013.821477

[bibr6-02698811251341371] Bossong MG Jager G Bhattacharyya S , et al. (2014) Acute and non-acute effects of cannabis on human memory function: A critical review of neuroimaging studies. Curr Pharm Des 20: 2114–2125. 23829369 10.2174/13816128113199990436

[bibr7-02698811251341371] Buysse DJ Reynolds CF 3rd Monk TH , et al. (1989) The Pittsburgh Sleep Quality Index: A new instrument for psychiatric practice and research. Psychiatry Res 28: 193–213. 2748771 10.1016/0165-1781(89)90047-4

[bibr8-02698811251341371] Chabrol H Ducongé E Casas C , et al. (2005) Relations between cannabis use and dependence, motives for cannabis use and anxious, depressive and borderline symptomatology. Addict Behav 30: 829–840. 15833585 10.1016/j.addbeh.2004.08.027

[bibr9-02698811251341371] Chen FF (2007) Sensitivity of goodness of fit indexes to lack of measurement invariance. Struct Equ Model Multidiscipl J 14: 464–504.

[bibr10-02698811251341371] Cicchetti DV (1994) Guidelines, criteria, and rules of thumb for evaluating normed and standardized assessment instruments in psychology. Psychol Assess 6: 284.

[bibr11-02698811251341371] Comrey AL Lee HB (2013) A First Course in Factor Analysis. East Sussex, UK: Psychology Press.

[bibr12-02698811251341371] Cooper ML (1994) Motivations for alcohol use among adolescents: Development and validation of a four-factor model. Psychol Assess 6: 117.

[bibr13-02698811251341371] Costello AB Osborne J (2005) Best practices in exploratory factor analysis: Four recommendations for getting the most from your analysis. Pract Assess Res Eval 10: 1–9.

[bibr14-02698811251341371] Couch D (2020) Left behind: The scale of illegal cannabis use for medicinal intent in the UK. Center for Medicinal Cannabis. Available at: https://thecmcuk.org/wp-content/uploads/2022/02/Left-Behind012020.pdf

[bibr15-02698811251341371] Davies-Owen J Christiansen P Roberts CA (2025) Associations between motivations for cannabis use and ‘the munchies’: Construct validity of the Cannabinoid Eating Experience Questionnaire. Subst Use Misuse 60: 20–27. 39279236 10.1080/10826084.2024.2403121

[bibr16-02698811251341371] Fabrigar LR Wegener DT MacCallum RC , et al. (1999) Evaluating the use of exploratory factor analysis in psychological research. Psychol Methods 4: 272.

[bibr17-02698811251341371] Faul F Erdfelder E Lang A-G , et al. (2007) G*Power 3: A flexible statistical power analysis program for the social, behavioral, and biomedical sciences. Behav Res Methods 39: 175–191. 17695343 10.3758/bf03193146

[bibr18-02698811251341371] Ghosh SK Burns CB Prager DL , et al. (2018) On nonparametric estimation of the latent distribution for ordinal data. Comput Statist Data Anal 119: 86–98.

[bibr19-02698811251341371] Gorelick DA Goodwin RS Schwilke E , et al. (2013) Tolerance to effects of high-dose oral Δ9-tetrahydrocannabinol and plasma cannabinoid concentrations in male daily cannabis smokers. J Anal Toxicol 37: 11–16. 23074216 10.1093/jat/bks081 PMC3584989

[bibr20-02698811251341371] Haug NA Padula CB Sottile JE , et al. (2017) Cannabis use patterns and motives: A comparison of younger, middle-aged, and older medical cannabis dispensary patients. Addict Behav 72: 14–20. 28340421 10.1016/j.addbeh.2017.03.006 PMC5492936

[bibr21-02698811251341371] Hoe SL (2008) Issues and procedures in adopting structural equation modelling technique. J Quant Methods 3: 76.

[bibr22-02698811251341371] Hu L Bentler PM (1999) Cutoff criteria for fit indexes in covariance structure analysis: Conventional criteria versus new alternatives. Struct Equ Model Multidiscipl J 6: 1–55.

[bibr23-02698811251341371] Hutcheson GD Sofroniou N (1999) The Multivariate Social Scientist: Introductory Statistics Using Generalized Linear Models. Washington DC: SAGE Publications.

[bibr24-02698811251341371] Ledesma RD Valero-Mora P (2007) Determining the number of factors to retain in EFA: An easy-to-use computer program for carrying out parallel analysis. Pract Assess Res Eval 12: 282–292.

[bibr25-02698811251341371] Lee CM Neighbors C Hendershot CS , et al. (2009) Development and preliminary validation of a comprehensive marijuana motives questionnaire. J Stud Alcohol Drugs 70: 279–287. 19261240 10.15288/jsad.2009.70.279 PMC2653613

[bibr26-02698811251341371] Loflin M Babson K Browne K , et al. (2018) Assessment of the validity of the CUDIT-R in a subpopulation of cannabis users. Am J Drug Alcohol Abuse 44: 19–23. 29058471 10.1080/00952990.2017.1376677

[bibr27-02698811251341371] Matali Costa J Simons J Pardo M , et al. (2018) Spanish version validation of the marihuana motives measure in a drug-consuming adolescent sample. Adicciones 30: 282–291. 29353295 10.20882/adicciones.947

[bibr28-02698811251341371] McNeish D (2018) Thanks coefficient alpha, we’ll take it from here. Psychol Methods 23: 412. 28557467 10.1037/met0000144

[bibr29-02698811251341371] Mindrila D (2010) Maximum likelihood (ML) and diagonally weighted least squares (DWLS) estimation procedures: A comparison of estimation bias with ordinal and multivariate non-normal data. Int J Digit Soc 1: 60–66.

[bibr30-02698811251341371] Newcomb MD Chou C-P Bentler PM , et al. (1988) Cognitive motivations for drug use among adolescents: Longitudinal tests of gender differences and predictors of change in drug use. J Counsel Psychol 35: 426.

[bibr31-02698811251341371] National Institute for Heath and Care Excellence (NICE) (2019) Cannabis-based medicinal products. NICE guidelines NG144. Available at: https://www.nice.org.uk/guidance/ng144 (accessed 5 May 2024). 31909928

[bibr32-02698811251341371] Nutt D Bazire S Phillips LD , et al. (2020) So near yet so far: why won’t the UK prescribe medical cannabis? BMJ Open 10: e038687. 10.1136/bmjopen-2020-038687 PMC7507889 32958492

[bibr33-02698811251341371] Office for National Statistics (ONS) (2023a). Drug misuse in England and Wales: Year ending March 2023. Office for National Statistics. https://www.ons.gov.uk/peoplepopulationandcommunity/crimeandjustice/articles/drugmisuseinenglandandwales/yearendingmarch2023 (accessed 1 July 2024)

[bibr34-02698811251341371] Office for National Statistics (ONS) (2023b) Released 2 February 2023, ONS website, article. Available at: https://www.gov.uk/government/statistics/substance-misuse-treatment-for-young-people-statistics-2021-to-2022/young-peoples-substance-misuse-treatment-statistics-2021-to-2022-report

[bibr35-02698811251341371] Pagano C Navarra G Coppola L , et al. (2022) Cannabinoids: Therapeutic use in clinical practice. Int J Mol Sci 23: 3344. 35328765 10.3390/ijms23063344 PMC8952215

[bibr36-02698811251341371] Pertwee RG Cascio MG (2014) Known Pharmacological Actions of Delta-9-Tetrahydrocannabinol and of Four Other Chemical Constituents of Cannabis that Activate Cannabinoid Receptors. Oxford, UK: Oxford University Press.

[bibr37-02698811251341371] Revelle W Zinbarg RE (2009) Coefficients alpha, beta, omega, and the glb: Comments on Sijtsma. Psychometrika 74: 145–154.

[bibr38-02698811251341371] Roberts CA Jager G Christiansen P , et al. (2019) Exploring the munchies: An online survey of users’ experiences of cannabis effects on appetite and the development of a Cannabinoid Eating Experience Questionnaire. J Psychopharmacol 33: 1149–1159. 31347452 10.1177/0269881119862526

[bibr39-02698811251341371] Sagar KA , et al. (2021) Assessing cannabis use disorder in medical cannabis patients: Interim analyses from an observational, longitudinal study. Cannabis 4: 47. 37287530 10.26828/cannabis/2021.02.004 PMC10212242

[bibr40-02698811251341371] Schlag AK Baldwin DS Barnes M , et al. (2020) Medical cannabis in the UK: From principle to practice. J Psychopharmacol 34: 931–937. 32522058 10.1177/0269881120926677 PMC7436434

[bibr41-02698811251341371] Simons J Correia CJ Carey KB , et al. (1998) Validating a five-factor marijuana motives measure: Relations with use, problems, and alcohol motives. J Counsel Psychol 45: 265.

[bibr42-02698811251341371] Singh K Junnarkar M Kaur J (eds.) (2016) Measures of positive psychology. In: Development and Validation. Berlin: Springer.

[bibr43-02698811251341371] Svíženská I Dubový P Šulcová A (2008) Cannabinoid receptors 1 and 2 (CB1 and CB2), their distribution, ligands and functional involvement in nervous system structures—A short review. Pharmacol Biochem Behav 90: 501–511. 18584858 10.1016/j.pbb.2008.05.010

[bibr44-02698811251341371] Tabachnick BG Fidell LS Ullman JB (2013) Using Multivariate Statistics. Boston, MA: Pearson.

[bibr45-02698811251341371] United Nations Office on Drugs and Crime (UNODC) (2019) World Drug Report 2019. United Nations. Available at: https://wdr.unodc.org/wdr2019/

[bibr46-02698811251341371] Velicer WF Eaton CA Fava JL (2000) Construct explication through factor or component analysis: A review and evaluation of alternative procedures for determining the number of factors or components. In: Helmes E Goffin RD (eds.) Problems and solutions in human assessment: Honoring Douglas N. Jackson at seventy. Berlin, Germany: Springer US, pp. 41–71.

[bibr47-02698811251341371] White HR Labouvie EW Papadaratsakis V (2005) Changes in substance use during the transition to adulthood: A comparison of college students and their noncollege age peers. J Drug Issues 35: 281–306.

[bibr48-02698811251341371] Yang KH Kaufmann CN Nafsu R , et al. (2021) Cannabis: An emerging treatment for common symptoms in older adults. J Am Geriatr Soc 69: 91–97. 33026117 10.1111/jgs.16833 PMC8962510

[bibr49-02698811251341371] Zhang X Noor R Savalei V (2016) Examining the effect of reverse worded items on the factor structure of the need for cognition scale. PLoS One 11: e0157795. 10.1371/journal.pone.0157795 PMC4909292 27305001

[bibr50-02698811251341371] Zvolensky MJ Vujanovic AA Bernstein A , et al. (2007) Marijuana use motives: A confirmatory test and evaluation among young adult marijuana users. Addict Behav 32: 3122–3130. 17602842 10.1016/j.addbeh.2007.06.010 PMC2213904

