# Supplementary material for: Development and validation of the Comprehensive Cannabis Motives Questionnaire (CCMQ)
Source: J Psychopharmacol. 2025 Jun 19;39(7):703–14. doi: 10.1177/02698811251341371 (PMC12267866; doi:10.1177/02698811251341371)
Supplement: sj-docx-1-jop-10.1177_02698811251341371 – Supplemental material for Development and validation of the Comprehensive Cannabis Motives Questionnaire (CCMQ) [file sj-docx-1-jop-10.1177_02698811251341371.docx]

**Results**

A one-way analysis of variance (ANOVA) was conducted to examine the effect of cannabis use recency on the CCMQ subscale scores. The subscales included Sleep, Medicinal, Social, High, Coping, Conform, Creative, and Food. Cannabis use recency had four levels: 24 Hours, Last Week, Last Month, and Last Three Months. Where significant main effects were observed, post-hoc pairwise comparisons were conducted using t-tests with Bonferroni correction.

A one-way analysis of variance (ANOVA) was conducted to investigate whether cannabis use recency (24 Hours, Last Week, Last Month, Last Three Months) influenced scores across the eight CCMQ subscales. Where significant main effects were found, pairwise comparisons were performed using Bonferroni-corrected t-tests.

For the Sleep subscale, the ANOVA was significant, *F*(3, 646) = 25.39, *p* < .001. Post-hoc tests revealed that individuals who had used cannabis in the last 24 hours reported significantly higher scores compared to those who had used in the last week (*p* = .001), last month (*p* < .001), and last three months (*p* < .001). Scores for the last week and last month groups did not differ significantly (*p* = 1.000), but both reported higher sleep-related motives than the last three months group (*p* <.001, and *p* = .056, respectively).

For the Medicinal subscale, there was a significant main effect, *F*(3, 646) = 46.22, *p* < .001. The 24 Hours group again reported significantly higher scores than the Last Week (*p* < .001), Last Month (*p* < .001), and Last Three Months (*p* < .001) groups. No significant difference was found between the Last Week and Last Month groups (*p* = 1.000), but both scored significantly higher than the Last Three Months group (*p* = .001 and *p* = .056, respectively).

No significant differences were found for the Social subscale, *F*(3, 646) = 1.46, *p* = .225, or for the High subscale, *F*(3, 646) = 1.09, *p* = .352.

For the Coping subscale, the ANOVA was significant, *F*(3, 646) = 19.33, *p* < .001. Pairwise comparisons indicated that scores were significantly higher for the 24 Hours group compared to the Last Month (*p* = .001) and Last Three Months (*p* < .001), but not significantly different from the Last Week group (*p* = .124). Additionally, the Last Week group scored significantly higher than the Last Three Months group (*p* < .001), though not compared to the Last Month group (*p* = .832). The Last Month group also scored higher than the Last Three Months group (*p* = .011).

The Conform subscale also yielded a significant main effect, *F*(3, 646) = 29.37, *p* < .001. The 24 Hours group had significantly higher scores than both the Last Month (*p* < .001) and Last Three Months (*p* < .001) groups, but not the Last Week group (*p* = .098). The Last Week group also scored higher than both the Last Month (*p* < .001) and Last Three Months (*p* <.001), while the Last Month and Last Three Months groups did not differ significantly (*p* = 1.000).

For the Creative subscale, a significant main effect was found, *F*(3, 646) = 11.34, *p* < .001. Post-hoc tests showed higher scores in the 24 Hours group than in the Last Week (*p* = .013), Last Month (*p* = .001), and Last Three Months (*p* < .001) groups. The Last Week group did not significantly differ from the Last Month (*p* = 1.000) or Last Three Months (*p* = .205), and no difference was observed between the Last Month and Last Three Months groups (*p* = 1.000).

Finally, for the Food subscale, the ANOVA revealed a significant effect, *F*(3, 646) = 13.99, *p* < .001. Scores were significantly higher in the 24 Hours group compared to the Last Three Months group (*p* < .001), but not compared to the Last Week (*p* = 1.000) or Last Month (*p* = .116) groups. The Last Week group scored significantly higher than the Last Three Months group (*p* < .001), and the Last Month group also reported higher scores than the Last Three Months group (*p* = .004).
